# Influence of copper content on the electrocatalytic activity toward methanol oxidation of Co_χ_Cu_y_ alloy nanoparticles-decorated CNFs

**DOI:** 10.1038/srep16695

**Published:** 2015-11-16

**Authors:** Zafar Khan Ghouri, Nasser A. M. Barakat, Hak Yong Kim

**Affiliations:** 1 Advanced Materials Institute for BIN Convergence, Department of BIN Convergence Technology, Chonbuk National University, Jeonju 561-756, Republic of Korea; 2 Department of Organic materials & Fiber Engineering, Chonbuk National University, Jeonju 561-756, Republic of Korea; 3 Department of Chemical Engineering, Faculty of Engineering, El-Minia University, El-Minia, Egypt

## Abstract

In this study, CoCu alloy nanoparticles-incorporated carbon nanofibers are introduced as effective non precious electrocatalyst for methanol oxidation in alkaline medium. The introduced electrocatalyst has been synthesized by simple and effective process; electrospinning. Typically, calcination, in nitrogen atmosphere, of electrospun nanofibers composed of cobalt acetate, copper acetate and poly (vinyl alcohol) leads to form carbon nanofibers decorated by CoCu nanoparticles. The nanofibrous morphology and alloy structure have been confirmed by SEM, TEM and XRD analyses. Investigation of the electrocatalytic activity indicates that copper content has strong influence, the alloy nanoparticles having the composition Cu_5%_Co_95%_ showed distinct high performance; 100 times higher than other formulations. Overall, the introduced study revealed the veil about the distinct role of copper in enhancing the electrocatalytic activity of cobalt-based materials.

The world is facing a rapidly increasing demand for sustainable energy. The global climate is threatened by anthropogenic changes, cohort of power without release of poisonous gases is crucial in existing scenario. The green technologies implemented for power generation include the use of air and water. However such technologies are in adequate to accomplish the existing power requirement^1^,^2^,^3^,^4^,^5^. Due to high consumption of energy subsequent from advances in technology as well as growing in population, additionally they started in depletion.

The emergence of fuel cell technology has created a new tool for the generation of clean, high competence substitute energy for humans.

The effectiveness of the fuel cells depends on the catalytic activity of the catalysts, based on the idea that substituting conventional fuel with methanol as a fuel can subsequently decrease greenhouse gas emission and smog pollution and it is a liquid which can be simply stored and handled.

Methanol is also easier to supply to the community using existing infrastructure. It is also can be produced from agricultural biomass and is considered a renewable energy^2^,^3^,^6^. Direct methanol fuel cells (DMFCs) are used as an alternative to petroleum driven vehicle^7^,^8^. So far, most of research work on fuel cells in the recent years focused on direct methanol and ethanol fuel cells. Commonly, the elementary procedures of DMFCs comprise methanol oxidation and oxygen reduction on precious metal catalysts.

The research and development of new catalysts to swap the rare platinum to reduce the overall cost of fuel cells is ongoing in this area. The novel electronic structure of the transition metals alloys strongly enhances the electrocatalytic activity; moreover carbon support can distinctly improves the performance due to the adsorption capacity.

At present, almost all pre-commercial low temperature fuel cells use Pt-based electrocatalysts for methanol electrooxidation. However, the high price and the limited presence of Pt prevent it from further commercialization. Furthermore the manufacturing cost is relatively high which constrain their wide applications, moreover it is well known that Pt surface can be easily poisoned by CO or HCO species leading to the rapid loss of the catalytic activity due to intermediate product which blocks the active sites of Pt which is another real problem facing most of the Pt based electrocatalyst^2^,^3^,^9^,^10^.

Therefore, great efforts have been made to investigate Pt-free electrocatalysts having the same competence to overcome the high cost of noble metals. Compared to the precious metals, transition metals are profuse, very inexpensive and stress-free to be produced, so they are used as substitutes for noble metals in various applications with high effectiveness. The profile, structure, and particle size of the transition metals have a straight effect on the catalytic performance of metals and electrocatalytic applications. The high surface area of metals nanostructure has improved the performance of catalytic reaction rate in the fuel cells^3^,^11^,^12^,^13^,^14^.

Among the investigated transition metals, nickel, copper and cobalt are the most popular metals due to their high proofed activity^5^,^15^,^16^,^17^. However, recently some reports were introduced about utilizing cobalt as the main component in effective electrocatalysts^2^,^18^,^19^. Alongside cobalt, copper based material also illustrate well-thought-of performance as an electrocatalyst^2^,^20^,^21^. Due to the synergetic impact and new electronic structure, the alloy composition reveals good performance as electrodes^15^,^22^. For case, MnCu alloy shows a much improved electrochemical activity for the oxidation of glucose in alkaline media in comparison to that of the pure Cu electrode^23^,^24^,^25^. Because of the adsorption capability of carbon, it was exploited to improve the electrocatalytic activities for numerous electrodes. Carbon materials have played important roles for generation of energy in different forms; recently, many kinds of carbon materials are employed in electrooxidation such as three-dimensional graphene network (3DGNs)^26^ and carbon cloth^27^.

Generally, among the reported nanostructures, nanofibers have gained much prominence in the recent year due to the high surface to volume ratio, better mechanical properties, and easy controllable and novel physical properties. Accordingly, the nanofibers present themselves as an outstanding applicant for a verity of potential applications such as insulations, garments, wipes, personal care, composites, barriers, filtration, medical, and energy storage. Furthermore, special properties of nanofibers make them suitable for wide range of high-tech applications such as capacitors, transistors, aerospace, drug delivery system, solar cell, information technology and fuel cells. Among several methods for nanofibers production, electrospinning is the most widely used technique due to simplicity, high yield, effectiveness and low cost aspects^2^,^3^,^4^,^28^. Currently, carbon nanofibers (CNFs) are widely used in many fields such as hydrogen energy^29^,^30^, electrochemical capacitors (EDLCs)^31^, lithium-ion rechargeable batteries (LIBs)^32^, supercapacitor^33^, and fuel cells^34^. Poly(vinyl alcohol) (PVA) is a semi-crystalline compound with comparatively high carbon content (ca. 54.5%), and easily splits hydroxyl groups in the polymer chain make PVA favorable for use as a precursor for the production of the carbonaceous materials, however low yield is the main constrain. The decomposition of PVA at temperatures slightly higher than its melting point is the discovered main reason for the low carbonization yield. Therefore, the researchers are trying to enhance the thermal stability of the PVA. Dehydration of PVA from 100 to 290 °C under tension in a mixed gas atmosphere induces the thermal stability required for carbonization^35^. Preoxidation or subsequent dehydration has also been employed as a PVA stabilization process^36^. Iodine acts as a good stabilizer of PVA and promotes dehydrogenative polymerization during carbonization^37^. Recently, it was reported that cobalt strongly enhances the graphitization of PVA^38^,^39^.

In this study, Co_x_Cu_y_ alloy nanoparticles incorporated in carbon nanofibers were investigated as a non-precious catalyst for methanol electrooxidation. Full study including the influence of the metallic nanoparticles composition and their loading was achieved. The results indicated that Cu content in the metallic alloy nanoparticles has distinct influence on the electrocatalytic activity.

## Results and Discussion

### Characterization of CoCu-decorated CNFs

**Figure 1:**
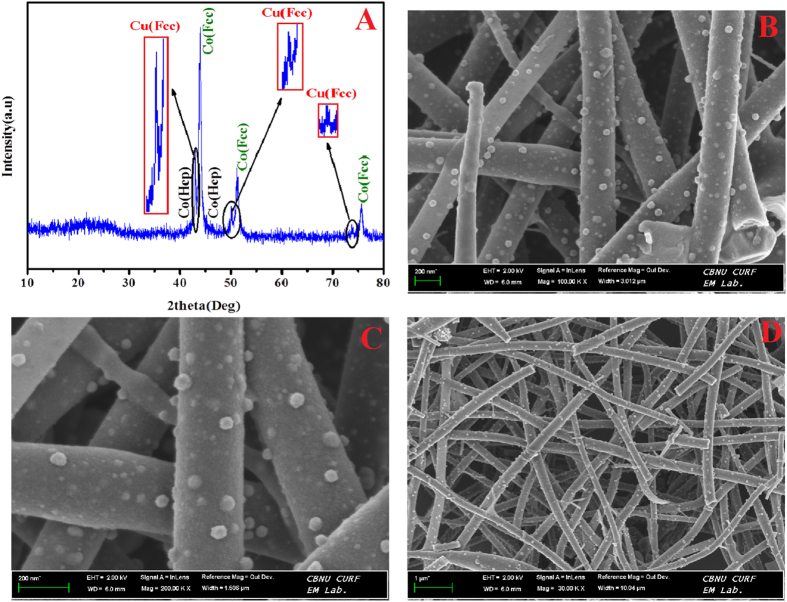
(**A**) XRD pattern (**B**–**D**) FESEM images of Cu_5%_Co_95%_-CNFs nanofibers after calcination of electrospun mats prepaing from sol-gel having 0.05 and 0.95 g of CuAc and CoAc, respectively at 900 °C for 7 h in nitrogen atmosphere.

Figure 1(A) shows the XRD patterns for the sintered nanofibers prepared from electrospun solution having 0.05 and 0.95 g CuAc and CoAc, respectively (Cu_5%_Co_95%_-CNFs); the symbols show the positions of XRD peaks of Co-FCC (JCPDS #15-0806), Co-HCP (JCPDS# 05-0727) and Cu (JCPDS# 3-2838). As shown in XRD pattern the strong diffraction peaks of 2θ values of 44.35°, 51.65° and 75.95° corresponding to (111), (200) and (220) crystal planes, respectively indicate formation of Co-FCC crystals moreover Co-HCP could be also detected as the standard peaks indicating that the formation of this phase can be seen at 41.72°, 44.73° and 47.63° corresponding to crystal planes of (100), (002) and (101), respectively. The aforementioned two phases of Co usually coexist at room temperature and difficult to be separated from each other and these two are most common phases of Co^40^. The XRD analysis in Fig. 1(A) specifies that one solid peak and two much weaker ones exist at 43.3°, 50.4° and 74° (marked by black circles & arrows along with high magnification for the marked area) which are indexed as the (111), (200) and (220) crystal planes, respectively of Cu-FCC (JCPDS # 3-2838). It was freshly stated that the (111) and (110) crystals planes of copper had boosted catalytic activity to methanol oxidation in contrast to the (100) crystal plane of copper^41^. Figure 1(A) shows that the (111) crystal plane exposed completely, which is promising for the catalytic activity to methanol oxidation. It is noteworthy mentioning that the same XRD data were obtained with the other formulations.

Beside the polycondensation property of acetate ions, during calcination under inert environment this ion abnormally decomposes and producing strong reducing gases (CO and H_2_) which play key role in complete reduction for the salt resulting in formation pristine metals rather than metal oxides^42^,^43^. Briefly formation of pure Co was explained by the following reactions^42^


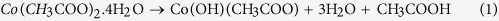



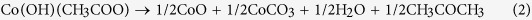



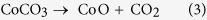



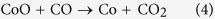


In case of copper acetate, it is difficult to understand by what mechanism the acetate group yields acetic acid as a product, but the process may be represented by the following stoichiometric equation^44^.


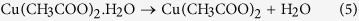



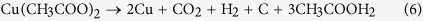


**Figure 2:**
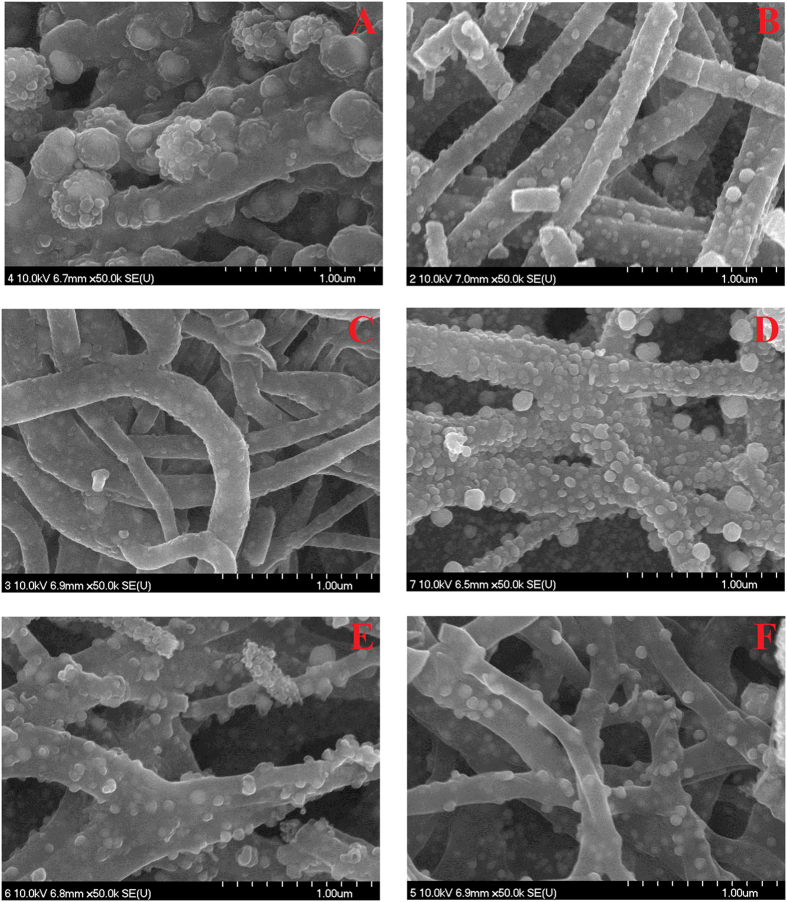
FESEM pattern for the obtained carbon nanofibers containing (A) Cu_0%_Co_100%_ (B) Cu_10%_Co_90%_ (C) Cu_15%_Co_85%_ (D) Cu_35%_Co_65%_ (E) Cu_65%_Co_35%_ and (F) Cu_100%_Co_0%_, nanoparticles.

Figure 1(B–D) shows the FESEM images of powder obtained from the electrospun mats containing 0.05 and 0.95 g CuAc and CoAc, respectively (Cu_5%_Co_95%_-CNFs). As shown, smooth and good morphology nanofibers were obtained. Moreover, the obtained nanofibers are decorated by nanoparticles which are expected to be Cu and/or Co metals. Figure 2 displays FE SEM images for nanofibers obtained from sol-gels having different CoAc/CuAc ratios, as shown almost all the powders demonstrate the nanofibrous morphology; however the best morphology was corresponding to CuAc content of 10 (Fig. 2B) and 15% (Fig. 2C).

**Figure 3:**
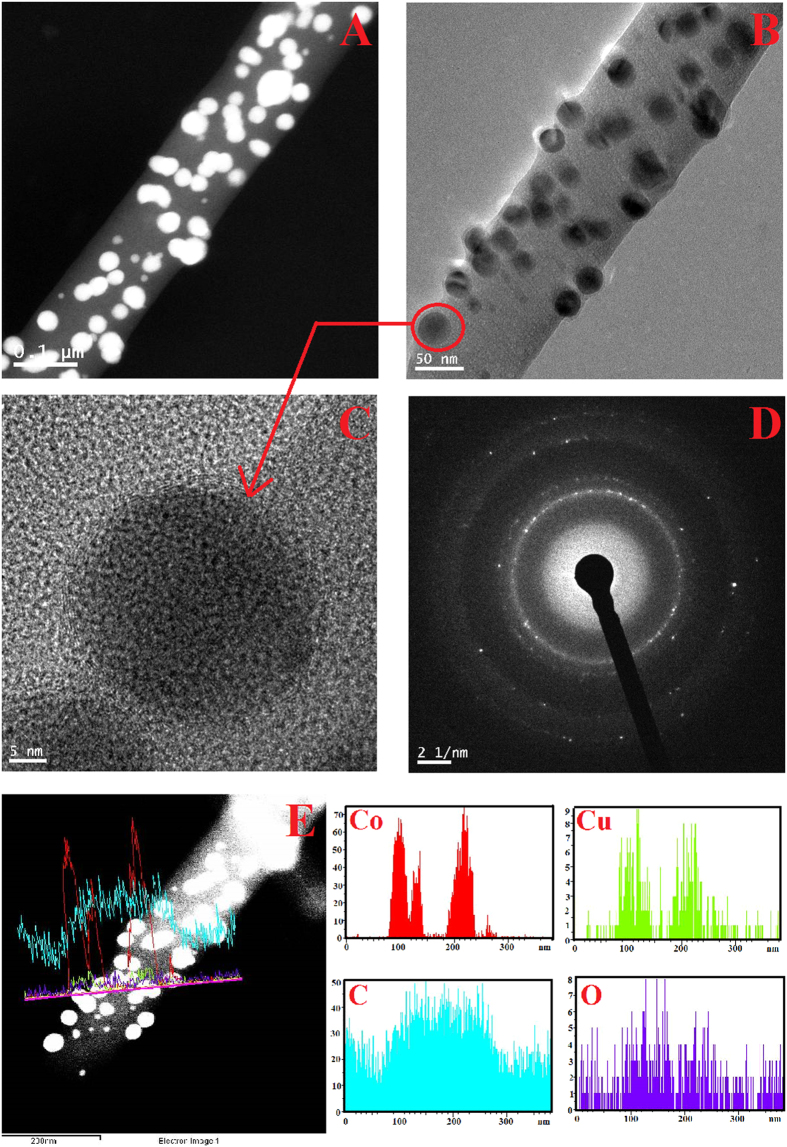
(**A**,**B**) TEM images (**C**) HRTEM image (**D**) SEAD image and (**E**) Line TEM EDX analysis of produced Cu_5%_Co_95%_-CNFs after calcination of produced nanofibers.

To examine the hypothesis of formation of bimetallic nanoparticles decorating carbon nanofibers; TEM analysis was carried. Figure 3(A,B) displays TEM images of the obtained sintered nanofibers from the electrospun mats containing 0.05 g CuAC (Cu_5%_Co_95%_ − CNFs). As shown, the nanofibers are ornamented by crystalline metallic nanoparticles distributed along with nanofibers which also supports the FESEM image (Fig. 1(B–D). Figure 3(C) displaying HR TEM image shows that the attached nanoparticles have good crystallinity (red arrow) which indicates that these nanoparticles compose of Co and/or Cu. Moreover the main nanofiber has an amorphous structure which refers to carbon. The selected area electron diffraction pattern (SEAD) demonstrated in panel D indicated good crystallinity for the metallic nanoparticles and simultaneously supports the XRD results.

To investigate the elemental distribution of the obtained nanofibers, TEM-EDX has been carried out; as shown in the Fig. 3(E), the attached metallic nanoparticles have different in sizes and also possess good crystallinity. Line EXD analysis was carried out at randomly selected line, the observed concentration profiles reveal that Co, Cu and C are detected along with chosen line. Interestingly, Co and Cu have almost the same elemental dissemination along with the nominated line which indicates alloy structure.

### Electrocatalytic activities

#### Influence of copper content

In contrast to the noble metals, the transition metals-based electrocatalyst own their activity from an active layer formed on the surface; e.g. NiOOH in the case of nickel. This active layer can be synthesized by sweeping the surface using multiple CV cycles in KOH (in case of utilizing in alkaline medium^45^,^46^,^47^.

**Figure 4:**
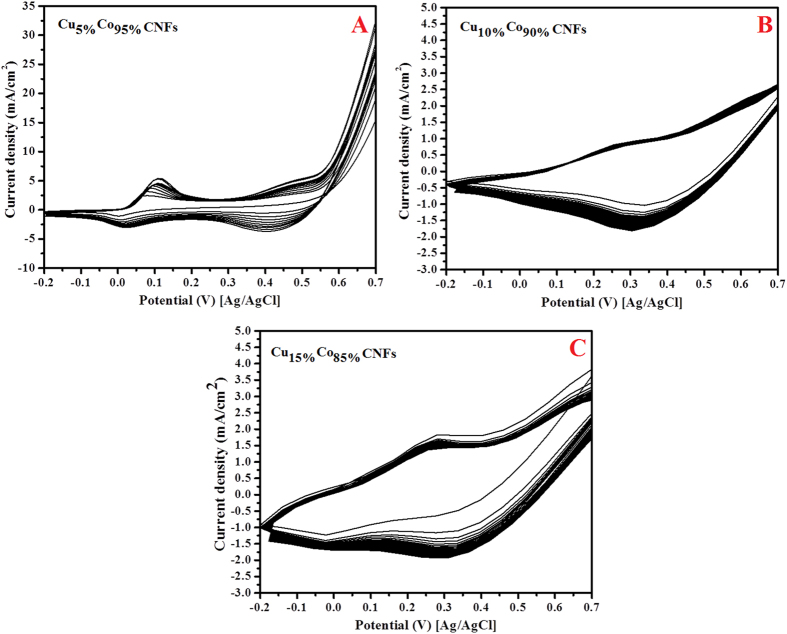
Cyclic voltammograms of the carbon nanofibers containing (A) Cu_5%_Co_95%_ (B) Cu_10%_Co_90%_ and (C) Cu_15%_Co_85%_ formulations in 1 M KOH. Scan rate 100 mV/s.

The cyclic voltammetric behaviors (in 1 M KOH solution) of the introduced CoCu-decorated CNFs with different Cu contents are introduced in Fig. 4A–C. Polarization was started by a potential scanning at a scan rate of 100 mVs^−1^ from 800 mV to −200 mV (vs. Ag/AgCl reference electrode) in the cathodic direction and then the scan was reversed in the anodic direction back to 800 mV.

As shown in Fig. 4A, the behavior of the Cu_5%_Co_95%_-CNFs nanofibers, two pairs of redox peaks can be observed. On the other hand, as shown in Fig. 4(B,C), increasing the copper content in the electrospun solutions led to almost disappearing of these redox peaks. Moreover, there is considerable difference in the corresponding current densities of the three formulations; Cu_5%_Co_95%_-CNFs sample reveals relatively higher values. Appearing of redox peaks is an important finding as it can be considered as an indicator for good electrocatalytic activity.

**Figure 5:**
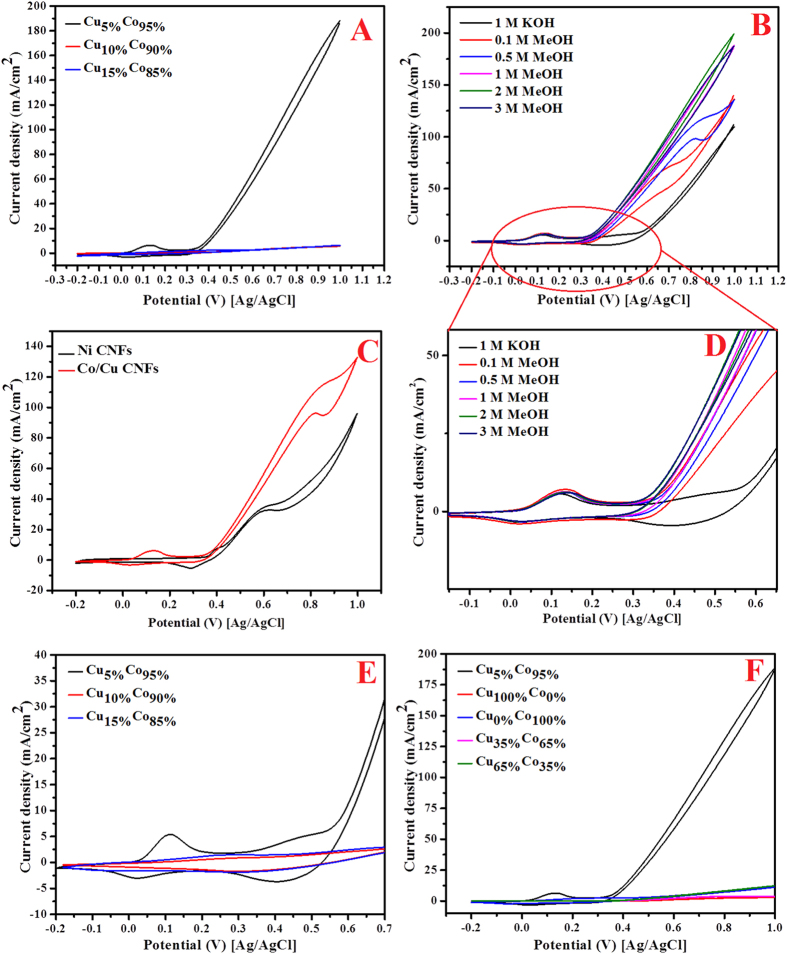
(**A**) Study the influence of Cu percentage on the electrocatalytic activity of the prepared CNFs containing (Cu_5%_Co_95%_, Cu_10%_Co_90%_ and Cu_15%_Co_85%_) toward methanol (3 M in 1 M KOH) oxidation (**B**) CV for the Cu_5%_Co_95%_-CNFs at different concentration of methanol (**C**) Performances of Cu_5%_Co_95%_ and Ni- decorated CNFs as electrocatalysts for(0.5 M in 1 M KOH) methanol oxidation (**D**) high magnification to show the onset potential (**E**) CV for 25 cycles during the activation process in presence of 1 M KOH (Scan rate 100 mV/s) for the carbon nanofibers containing Cu_5%_Co_95%_, Cu_10%_Co_90%_ and Cu_15%_Co_85%_ nanoparticles and (**F**) Study the influence of various percentages of Cu and Co on the electrocatalytic activity of the prepared CoCu-CNFs toward methanol oxidation (3 M in 1 M KOH) at scan rate 50 mV/s at 25 °C.

To properly evaluate the electrocatalytic activity of the activated Cu_x_Co_y_-decorated CNFs, methanol electrooxidation was carried out in 1M KOH solution. Figure 5(A) displays the activities in presence of 1 M methanol (in 1 M KOH). As shown, the copper content has very strong influence on the obtained current densities. Surprisingly, the nanofibers obtained from electrospun solution having 5 wt% CuAc (Cu_5%_Co_95%_-CNFs) reveal current density (190 mA/cm^2^) 100 times more than the other formulations which comparatively have inconsiderable activities. This finding can be attributed to appearing of the clear redox peaks for this distinctly active formulation (Fig. 4A).

It is noteworthy mentioning that, among the transition metals, nickel is the most widely used due to its high activity especially in methanol electrooxidation. As shown in Fig. 5(C), the performance of the introduced material has been compared to Ni-decorated nanofibers. The results affirmed the activity of the introduced Co/Cu-decorated CNFs as the corresponding current density was higher as shown in the figure.

The optimum methanol concentration is a process parameter for every electrocatalyst. In other words, as water is a reactant in the methanol oxidation reaction, the optimum methanol concentration should be assigned for the introduced catalyst. Fig. 5(B) displays the influence of methanol concentration on the obtained current density of the best nanofibers observed in Fig. 5(C). As shown in the figure, the methanol concentration increase leads to the increase of the current densities which specifies methanol oxidation on the surface of the presented nanofibers. It can be observed in Fig. 5(B) the optimum methanol concentration of 2 M which is consistent with many reported electrocatalysts.

The onset potential is a significant indicator among the appealed parameters to prove the electrocatalytic activity. Generally, in alcohols electrooxidation, more negative onset potential shows high activity and less over potential. As shown in Fig. 5(D), the observed onset potential of the introduced nanofibers is ~310 mV (vs. Ag/AgCl), and the achieved assessment is relatively low compared to numerous reported materials^48^. Moreover, it is clear that the onset potential is independent on the methanol concentration as well as the alloy composition. This discovery further supports the electrocatalytic performance of the introduced nanofibers.

As shown in Fig. 5(E), the best decorated CNFs show cathodic peak at ~50 mV, this peak is almost at the same potential of the cathodic peak in the activation of this formulation (Fig. 4A) which is responsible about the catalyst regeneration^49^. Therefore, the observed high negative current density in the cathodic direction can be attributed to either oxidation of the intermediate compounds during methanol oxidation in the anodic direction and/or reactivation of the surface. Both assumptions can be considered as acceptable reasons for the observed high activities.

It is known that the alloy structure does have electron configuration differs than the individual constituents which provides special physicochemical characteristics. Moreover, this electron configuration depends mainly on the composition. As a heterogeneous catalyst, the performance of the electrocatalyst in methanol oxidation depends on the electronic structure of the catalyst. From TEM EDX results (Fig. 3E), the formed Co/Cu nanoparticles possess an alloy structure. Accordingly, the influence of the composition of the CoCu NPs decorating the CNFs on the electrocatalytic activity has been investigated by synthesizing more CoCu-CNFs having more different compositions for the metallic nanoparticles. Typically, the electrospun solutions were adjusted to produce final CNFs decorated by metallic nanoparticles with composition of Cu_0%_Co_100%_, Cu_5%_Co_95%_, Cu_35%_Co_65%_, Cu_65%_Co_35%_, and Cu_100%_Co_0%_. Figure 5(F) displays the electrocatalytic performances in presence of 1 M methanol. As shown in the figure, pristine Co- and Cu- (Cu_0%_Co_100%_ and Cu_100%_Co_0%_, respectively) decorated CNFs almost do not have electrocatalytic activity toward methanol oxidation. Moreover, increasing the copper content in the formulations having nanoparticles with the composition of Cu_35%_Co_65%_ and Cu_65%_Co_35%_ has also distinct negative impact on the activity. Interestingly, the same formulation discovered before (Cu_5%_Co_95%_) still prominent as it reveals the maximum and incomparable activity to the other formulations.

**Figure 6:**
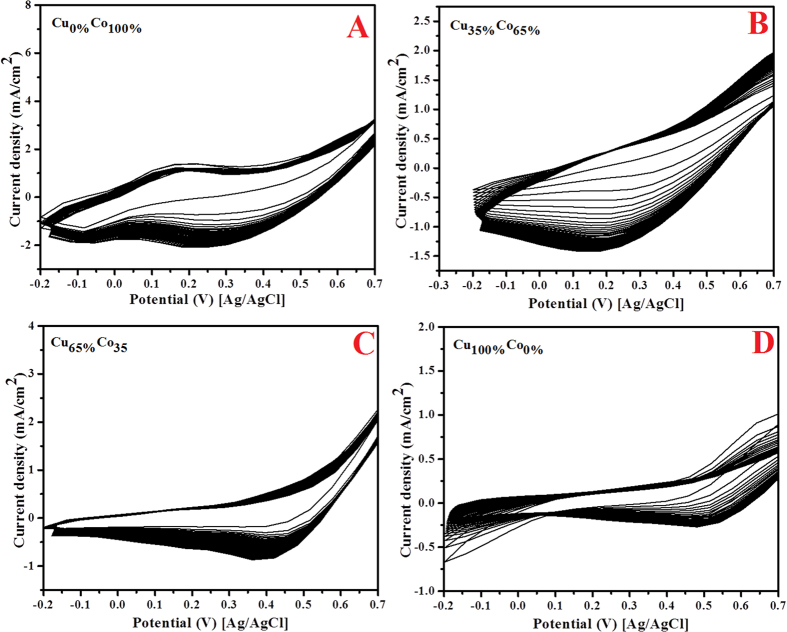
Cyclic voltammograms of the carbon nanofibers containing (**A**) Cu_0%_Co_100%_ (**B**) Cu_35%_Co_65%_ (**C**) Cu_65%_Co_35%_, and (**D**) Cu_100%_Co_0%_ nanoparticles. Scan rate 100 mV/s, 1 M KOH.

**Figure 7:**
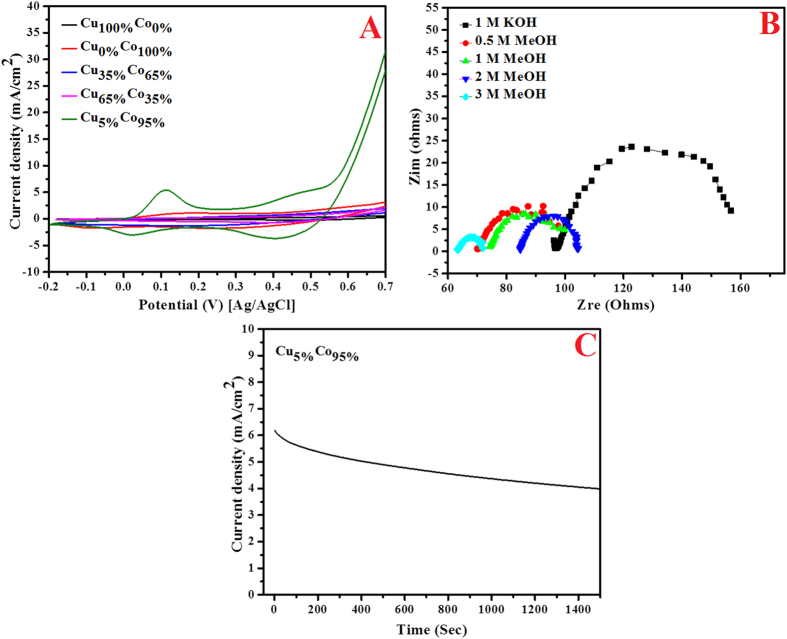
(**A**) CV for 50^th^ cycle of the activation process in presence of 1 M KOH (Scan rate 100 mV/s) for the carbon nanofibers containing Cu_0%_Co_100%_, Cu_35%_Co_65%_, Cu_65%_Co_35%_, Cu_5%_Co_95%_ and Cu_100%_Co_0%_ nanoparticles (**B**)Nyquist plot of Cu_5%_Co_95%_-CNFs at different concentrations of methanol and (**C**) Chronamperometery for Cu_5%_Co_95%_-CNFs electrode at *V* = 0.4 V vs. Ag/AgCl in methanol in alkaline medium (3 M methanol in 1 M KOH).

The distinctly big difference in the activity observed in Fig. 5(F), might be understood from the activation behaviors on the investigated formulations. Figure 6 displays the sweep cyclic voltogramms in presence of 1 M KOH for 50 cycles at scan rate 100 mV/s. Compared the best formulation (Fig. 4A), one cannot find characteristic redox peaks in the investigated formulations. Figure 7(A), displays comparison between the investigated alloys compositions compared to the best formulation, as shown the redox peaks in the optimum composition are clearly seen while almost no peaks could be found in the other prepared modified CNFs.

Overall, according to the obtained results, the observed distinguished electrocatalytic activity of the best formulation can be attributed to formation of ZOOH/Z(OH)_2_ on the surface of the *Z* alloy where *Z* refers to the optimum composition (Cu_5%_Co_95%_). Analogy to the transition metals, formation of the ZOOH layers can be clarified by the following reactions^50^


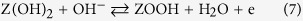


Where *Z*(OH)_2_ was synthesized on the surface of the *Z* alloy NPs due to its electron configuration which enhances formation of stable hydroxide layer. Therefore, in Fig. 4(A), increasing the number of potential sweeps resulting in progressive increase of current density values of the cathodic peak since of the entry of OH^−^ into the *Z*(OH)_2_ surface layer, which leads to the progressive formation of a thicker *Z*OOH layer analogous to the Z(OH)_2_/ZOOH transition^50^ which results in high catalytic activity. Accordingly, the mechanism of electrooxidation of methanol using the introduced modified nanofibers matches the introduced one on the preceding literature^44^,^48^,^51^. Briefly, in the first step of the methanol oxidation, the prepared catalyst (*Z*) adsorb methanol and partially release protons in the second and third step more protons released. Normally the *Z*_3_COH species decomposes to produce *Z* and protons in step 4^51^.

#### EIS Characterization

Electrochemical impedance spectroscopy (*EIS*) was performed to examine the methanol oxidation. Nyquist plots for the investigated electrode in different methanol concentrations (0, 0.5, 1, 2 and 3 M + 1.0 M KOH) are displayed in Fig. 7(B). Considering the Nyquist plot of the presented electrode, the acquired plot specifies that the current leads to capacitive charging of the double layers. In other words, it can be concluded that the cell has polarizable electrodes; this assumption is confirmed by the insignificant influence of increasing methanol concentration on the Nyquist plots as shown in the figure. In the Nyquist plot, the Faradic reaction (methanol oxidation) commonly showed by capacitive loop with a diameter almost corresponding to the charge transfer resistance (*R*_CT_). As shown in the figure lower charge transfer resistance is obtained at 3 M concentration of methanol.

Table 1 summarizes the values of the *R*_CT_ in Ω cm^2^ for the investigated electrode at different methanol concentrations. As shown, the charge transfer resistance inversely proportions to the methanol concentration which indicates good activity.

#### Chronoamperometry

Beside the sought-for high electrocatalytic performance, stability was another target from preparing of the alloy structure. Figure 7(C), shows the chronoamperogram of electrode recorded for nearly 1500 s at fixed potential (*E* = 0.4 V) in 3 M methanol + 1 M KOH at room temperature. As shown in the figure, there is an initial current drop, followed by a very slow decay. It is noteworthy mentioning that, only one working electrode has been used in all the electrochemical analysis. Multiple use of the electrode did not affect the performance which supports the good stability of the introduced catalyst. This figure supports the good stability of introduced catalyst which can be attributed to the alloy structure.

## Conclusion

Electrospinning technique can be utilized to produce smooth, beads free and good morphology nanofibers composed of poly(vinyl alcohol), copper acetate and cobalt acetate. Calcination of the resultant electrospun mats in nitrogen atmosphere leads to form carbon nanofibers decorated by CoCu bimetallic alloy. The electrocatalytic activity toward methanol electrooxidation mainly depends on the composition of the bimetallic CoCu alloy nanoparticles attaching the carbon nanofibers. Typically, the optimum composition is related to the bimetallic nanoparticles having 5 wt% Cu. The suggested non-precious electrocatalyst can be further improved to be more effectual for fuel cell applications.

## Method

### Materials

Copper (II) Acetate monohydrate (CuAc, 98%) and Cobalt (II) acetate tetra hydrate (CoAc, 98%) were bought from Showa chemicals co Ltd Japan and Junsei chemicals co Ltd Japan, respectively. Poly (Vinyl alcohol) PVA with a molecular weight 65000 g/mol was obtained from Aldrich, USA, Distilled water was used as solvent.

### Preparation of CoCu CNF’s

CoAc and CuAc aqueous solutions were firstly prepared by dissolving different amounts CuAc and CoAc (total amount of CoAc and CuAc was 1 g) in 3 ml of distilled water with 2 h stirring at room temperature and then mixed with 15 g PVA aqueous solution (10 wt%). Finally the mixture was stirred at 50 °C for 6 h to get see-through, clear and consistent mixture. The achieved sol-gel was electrospun at high voltage of 22 kV using DC power supply at room temperature with 65% relative humidity. The distance between needle tip (positive electrode) and rotating cylinder (negative electrode) was kept constant at 22 cm. The ready nanofiber mats were normally dried at room temperature for 12 h and then under vacuum for 24 h at 60 °C, lastly the dried nanofibers were calcined at 900 °C for 7 h in nitrogen atmosphere with heating rate of 2.0 °C/min.

### Sample characterization

The phase and crystallinity of the composite were characterized by X-ray diffract meter (XRD, Rigaku, Japan) with Cu-Kα (*λ* = 1.54056 Å) radiation over a range of 2Θ angle from 10° to 80°. The morphology of the products was observed by field-emission scanning electron microscopy (FESEM, Htachi S-7400, Japan) whereas the distribution of elements was measured using energy dispersive X-ray spectroscopy (EDX) analysis. While high resolution TEM images and selected area electron diffraction patterns were observed by JEOL JEM-2200FS transmission electron microscope (TEM) operating at 200 kV equipped with EDX (JEOL, Japan).

### Electrochemical measurements

Electrochemical measurements were conducted using VersaSTAT4 (USA) and conventional three electrode electrochemical cell at room temperature. An Ag/AgCl, Pt wire, and 3 mm glassy carbon were used as the reference, counter and working electrode, respectively. For the preparation of the catalyst electrode, a 2 mg of the synthesized alloy NFs were spread in a suspension of 400 μl propanol and 20 μl of a nafion solution under ultrasonic stirring for 1 h to ensure good dispersion. A 15 μl aliquot of the slurry was spread on the top of the sophisticated working electrode surface and dried at 80 °C for 30 min. The active zone of the utilized glassy carbon electrode was a circle with a diameter of 3 mm. Therefore, the active area was estimated (0.0713 cm^2^) and all the data was normalized to this area.

## Additional Information

**How to cite this article**: Ghouri, Z. K. *et al.* Influence of copper content on the electrocatalytic activity toward methanol oxidation of Co_χ_Cu_y_ alloy nanoparticles-decorated CNFs. *Sci. Rep.* **5**, 16695; doi: 10.1038/srep16695 (2015).

## Figures and Tables

**Table 1:** Charge Transfer resistance (*R*_CT_, Ω cm^2^) for CoCu CNFs at different methanol concentrations.

Methanol Concentration (M)	0.0	0.5	1	2	3
*R*_CT_, Ω cm^2^	11.06	6.95	6.91	7.20	4.90
